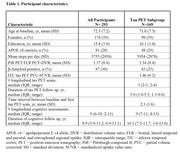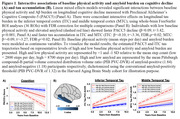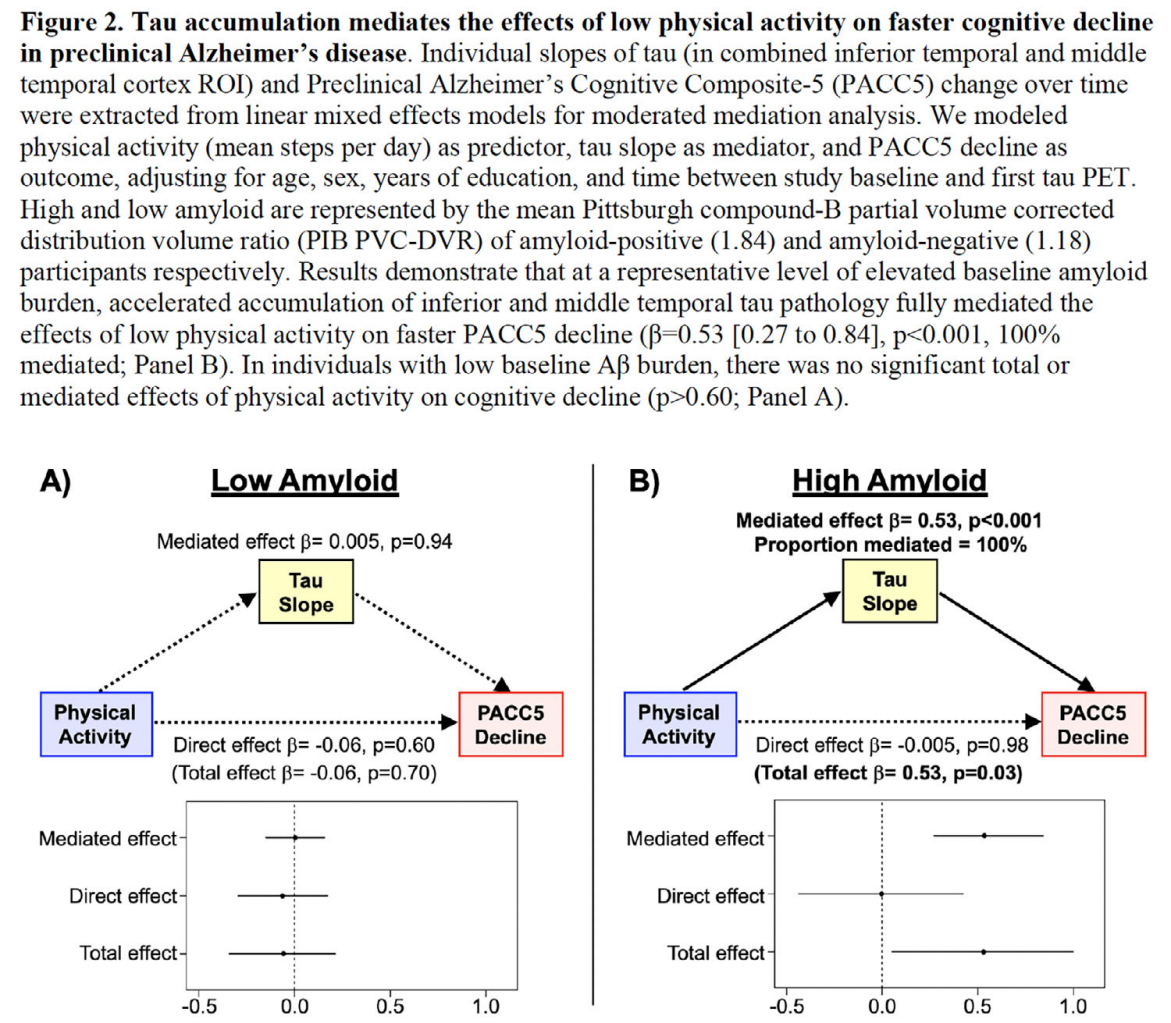# Accelerated Temporal Cortex Tau Accumulation Mediates the Effects of Low Physical Activity on Faster Cognitive Decline in Preclinical Alzheimer's Disease

**DOI:** 10.1002/alz.093936

**Published:** 2025-01-09

**Authors:** Wai‐Ying Wendy Yau, Dylan Kirn, Jennifer S Rabin, Michael J Properzi, Aaron P Schultz, Dorene M. Rentz, Keith A Johnson, Reisa A Sperling, Jasmeer P. Chhatwal

**Affiliations:** ^1^ Massachusetts General Hospital, Harvard Medical School, Boston, MA USA; ^2^ Massachusetts General Hospital, Boston, MA USA; ^3^ Harquail Centre for Neuromodulation, Sunnybrook Research Institute, Toronto, ON Canada; ^4^ Department of Neurology, Massachusetts General Hospital, Harvard Medical School, Boston, MA USA; ^5^ Athinoula A. Martinos Center for Biomedical Imaging, Harvard Medical School, Charlestown, MA USA; ^6^ Center for Alzheimer Research and Treatment, Department of Neurology, Brigham and Women's Hospital, Boston, MA USA; ^7^ Center for Alzheimer's Research and Treatment, Brigham and Women's Hospital, Harvard Medical School, Boston, MA USA; ^8^ Massachusetts General Hospital, Harvard Medical School, Department of Neurology, Boston, MA USA; ^9^ Brigham and Women's Hospital, Harvard Medical School, Boston, MA USA

## Abstract

**Background:**

Physically inactivity is associated with increased risk of dementia including Alzheimer’s disease (AD). Prior work from the Harvard Aging Brain Study (HABS) suggests that lower baseline physical activity in cognitively unimpaired individuals with elevated amyloid burden is associated with faster prospective cognitive decline. However, whether this detrimental effect on cognition in preclinical AD is mediated by accelerated tau pathology remains unclear.

**Method:**

We examined 293 baseline cognitively unimpaired older adults from HABS (Table 1). Baseline physical activity (mean steps per day) was measured using a waistband‐mounted pedometer (HJ‐720ITC; Omron Healthcare) worn over 7 consecutive days during waking hours. Using linear mixed effects models, we examined the interactive effects of baseline physical activity and amyloid PET burden (Pittsburgh Compound‐B) on longitudinal cognitive decline (Preclinical Alzheimer Cognitive Composite‐5 [PACC5]), adjusting for age, sex and education (APOE not significant). In a subset of 169 participants, we examined the interactive effects on longitudinal tau pathology (Flortaucipir PET) in 36 whole‐brain FreeSurfer cortical and limbic ROIs (FDR‐correction). We further investigated if the physical activity effects on PACC5 decline in preclinical AD are mediated by changes in tau.

**Result:**

In a larger sample of HABS participants with up to 13 years of cognitive follow‐up, we replicated the significant interaction between lower physical activity and elevated amyloid burden on faster prospective cognitive decline (ß=0.09, t=3.42, p<0.001; Figure 1A). We further demonstrated a novel interactive effect on tau pathology, where lower physical activity was associated with accelerated amyloid‐related tau accumulation in inferior temporal (ITC) and middle temporal (MTC) cortices (ITC: ß=‐0.10, t=‐3.36, FDR‐p=0.02; MTC: ß=‐0.09, t=‐3.27, FDR‐p=0.02; Figure 1B). Importantly, moderated mediation analyses revealed that accelerated tau accumulation fully mediated the effects of lower physical activity on faster cognitive decline in the setting of elevated amyloid (p<0.001, 100% mediated; Figure 2).

**Conclusion:**

Our findings provide strong support for promoting physical activity as a lifestyle intervention, in conjunction with anti‐amyloid therapy, to slow the onset and/or progression of early neocortical tau pathology and cognitive decline in preclinical AD.